# Human perception of self-motion and orientation during galvanic vestibular stimulation and physical motion

**DOI:** 10.1371/journal.pcbi.1012601

**Published:** 2024-11-18

**Authors:** Aaron R. Allred, Caroline R. Austin, Lanna Klausing, Nicholas Boggess, Torin K. Clark

**Affiliations:** Bioastronautics Laboratory, Smead Department of Aerospace Engineering Sciences, University of Colorado–Boulder, Boulder, Colorado, United States of America; Chinese Academy of Sciences, CHINA

## Abstract

Galvanic vestibular stimulation (GVS) is an emergent tool for stimulating the vestibular system, offering the potential to manipulate or enhance processes relying on vestibular-mediated central pathways. However, the extent of GVS’s influence on the perception of self-orientation pathways is not understood, particularly in the presence of physical motions. Here, we quantify roll tilt perception impacted by GVS during passive whole-body roll tilts in humans (N = 11). We find that GVS systematically amplifies and attenuates perceptions of roll tilt during physical tilt, dependent on the GVS waveform. Subsequently, we develop a novel computational model that predicts 6DoF self-motion and self-orientation perceptions for any GVS waveform and motion by modeling the vestibular afferent neuron dynamics modulated by GVS in conjunction with an observer central processing model. This effort provides a means to systematically alter spatial orientation perceptions using GVS during concurrent physical motion, and we find that irregular afferent dynamics alone best describe resultant perceptions.

## Author summary

Vestibular-mediated deficits are a primary concern for astronauts, aviators, and individuals on Earth. These deficits include loss of spatial orientation, impaired postural control and locomotion, loss of fine motor control, and motion sickness. Further, vestibular dysfunction negatively impacts aging adults, affecting more than a third of US adults over 40. One promising means of reducing these vestibular-mediated deficits is through applied Galvanic Vestibular Stimulation (GVS). GVS affects vestibular sensory information through current applied via skin-surface electrodes, most commonly situated over the mastoid processes, behind the ears. However, the full extent that GVS may be utilized for these purposes has remained unknown. Here, we empirically quantify the influence of GVS on roll perception during dynamic, passively-applied, whole-body roll motions. To extend our understanding further, we develop and validate a computational model from this data that can predict spatial orientation perceptions across any applied current or passively applied physical motion profile.

## Introduction

Our ability to control and maintain orientation is largely reliant on vestibular sensory information. Critical for many functions within the terrestrial environment, the vestibular system transduces information of head motion which is centrally processed to produce reflexive gaze and posture stabilizations as well as perceptions of self-motion and self-orientation **[1]**. Over the last century, human presence in air and space environments has increased the demands of vestibular sensory processing. Pilots experience lethal spatial orientation misperceptions [2, 3]; astronauts commonly exhibit debilitating sensorimotor deficits [4, 5]; and both populations often experience severe motion sickness symptoms [6, 7]. Outside of these domains, vestibular dysfunction negatively impacts more than a third of US adults over 40 and can result in instability [8], reduced motion perception [9], blurry vision, and vertigo. An emergent technology, galvanic vestibular stimulation (GVS) may help remedy peripheral and central vestibular deficiencies through transcutaneous electrical stimulation as well as an improved understanding of vestibular-mediated neural substrates.

For use clinically, recent investigations of GVS have explored ameliorating vestibular and balance-associated disorders, spanning from treating vestibulopathy [10] to symptoms of Parkinson’s disease [11] and stroke [12]. Peripheral electrical augmentation of vestibular sensory information has been leveraged to partially correct vestibular ocular reflexes through invasive methods (i.e., the vestibular prosthesis), and knowledge acquired through GVS research enhances the implementation of these techniques. GVS has also been explored for improving and modifying the perception of self-motion, critical for navigating in the air and space domains. Applications include use as an alternative display modality [13], enhancing simulator immersion [14, 15], providing a post-spaceflight sensorimotor analog [16, 17], and potentially helping mitigate motion sickness [18–20]. However, the full utility of GVS is contingent on our ability to predict central processing (e.g., for spatial orientation perception), in the presence of both physical motions and artificial GVS current application.

Recent works have begun to explore GVS’s holistic impact on human perception of self-motion and self-orientation. Schneider et al. [21] measured the required GVS amplitude to produce sub-threshold perceptions of tilt during sinusoidal motion and induce a perception of translation. In a quantitative evaluation of perception, Niehof et al. [22] measured roll tilt in stationary participants during varying levels of direct current GVS. To explain sensations of rotation and translation from GVS, Khosravi-Hashemi et al. [23] employed a model of central processing of vestibular information and qualitatively evaluated perceptions of rotation and translation in stationary participants. In another modeling effort, Chen et al. [24] examined the relationship between electrical GVS stimuli and canal signals without central processing. Despite these advances, it remains undetermined to what extent peripheral modulation of the afferent neurons via GVS, in combination with contributions from physical stimuli, results in modified human self-orientation and self-motion percepts. Without a validated, comprehensive model, we cannot use GVS to make predictions in new scenarios, particularly those with physical motion, or drive desired perceptual outcomes.

**Fig 1:**
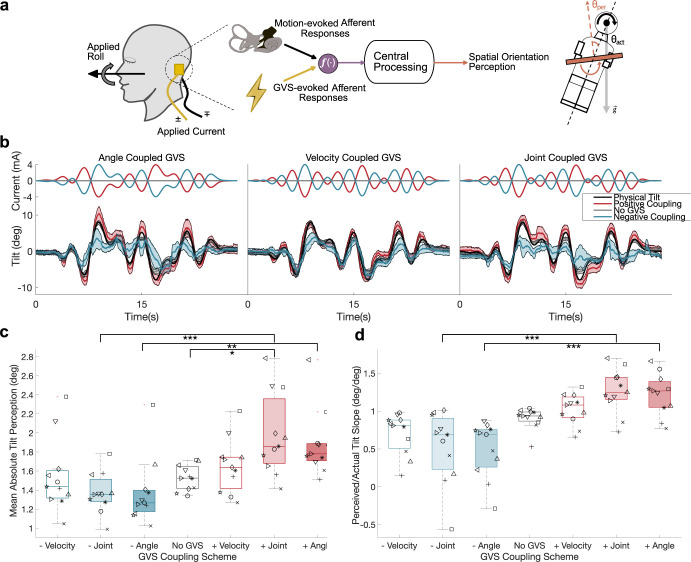
a, During dynamic GVS and applied whole-body roll, continuous perceptions of roll tilt were collected in human participants (N = 11) using a subjective haptic horizontal task. **b,** Across trials, physical tilt was provided to participants with three GVS directional couplings: positively coupled (red), negatively coupled (blue), and No GVS (gray). Positive current corresponds to left cathode right anode current direction to maintain consistency with existing literature. Shaded regions correspond to SEM bounds around the mean for the sample population. Perceptual results are shown for a single motion and single direction, and more comprehensive results are shown in Fig 4C and 4D, Metric computations and resultant distributions are provided for the two scalar metrics. Box plots report medians and quartiles. Individual participant identifiers (markers) are consistent throughout. **c,** Results for the Mean Absolute Tilt Perception. **d,** Results for the Perceived/Actual Tilt Slope.

Accordingly, we first measured perceptions of roll tilt in human participants in the presence of both dynamic physical motion stimuli and dynamic GVS currents using a standard psychophysical task (Fig 1A), furthering earlier empirical examinations [21, 22]. Next, we advance upon previous modeling efforts [23, 24] to explain the modified dynamic perceptions of tilt evoked by GVS via a systems neuroscience understanding of vestibular sensory processing. Specifically, a computational model of human perception due to both physical and GVS-induced virtual motion, based on the “observer model” for spatial orientation perception during passive motions [25–27] is developed, with afferent activation dynamics [28, 29] informing the computational model’s architecture. Ultimately, we present an observer model of human self-motion perception augmented to capture the influence of GVS on vestibular sensory afferents in humans, trained and validated across multiple paradigms, which provides quantitative predictions of self-motion and self-orientation in the presence of any GVS waveform and any physical motion for the first time.

## Results

### Perception of roll-tilt during physical motions systematically depends on GVS waveforms and physical motion profiles

We empirically examined roll tilt perceptions during GVS and passive whole-body roll tilt in the absence of other sensory cues (Fig 1A). This empirical assessment enabled both the characterization and quantification of GVS effects in the presence of naturalistic physical motion (0.07–0.36Hz), across comparable frequencies of GVS current at tolerable current amplitudes (+/-4**mA** peak current). GVS current was directionally coupled either a) positively (anticipating an amplification of roll-tilt perceptions) or b) negatively (anticipating an attenuation of roll-tilt perceptions) to a physical characteristic of the motion profile: either 1) the roll tilt angle, 2) roll tilt velocity, or 3) an equal combination of both (jointly coupled). In addition, a sham condition (No GVS), yields 7 total coupling schemes (Fig 1B). Each of these was assessed with 3 different physical motion profiles comprised of dynamic (sum of sinusoids ranging from 0.07 to 0.36 Hz), head-centered whole-body roll tilt (Fig 1B).

Our experimental results first revealed differences in perceptual reports that were dependent on both the tilt profile and GVS waveforms, confirming our first experimental hypothesis that perceptions of roll tilt during whole-body physical roll tilts depend on the specific physical motion and the GVS waveform sensed by the vestibular organs. Two scalar metrics were utilized to quantify how our independent variables affected perceptions of roll tilt as captured by a subjective haptic horizontal (SHH) task: Mean Absolute Tilt Perception (which captures the amplitude of tilt perception, agnostic to physical tilt) and the slope of perceived tilt versus actual tilt (Perceived/Actual Tilt Slope, which compares tilt perception to the physical tilt at that moment). These metrics provide complimentary means of characterizing roll tilt perception amplification/attenuation across experimental conditions, agnostic to underlying central processes. For both of the metrics, a 3-factor repeated measures ANOVA across coupling scheme (7 conditions), physical motion profile (3 conditions), and initial motion direction (mirrored tilts; 2 conditions) revealed significant differences for coupling schemes (F (6,18) > 6.35; p< 0.001) for both metrics and significant differences between motion profiles (F(2,6) = 6.36; p = 0.033) for Mean Absolute Tilt Perception. There were no differences for right or left starting directions and no interaction effects (p>0.22), revealing that left/right mirrored repetitions of motions did not affect perceptions, as expected.

### Amplification and attenuation of roll tilt perception is achieved through selective directional current coupling

Next, we assessed the directional dependency of GVS on over and under-estimation of tilt. Qualitatively, the effect of GVS in each of the three unique motion profiles and seven unique coupling schemes revealed consistently amplified perceptions of physical tilt during positive directional couplings and attenuated perceptions during negative directional couplings (Fig 1B). Conversely, perceptions during the No GVS condition paralleled the temporal dynamics of actual tilt. To quantitatively evaluate differences in directional GVS couplings across physical couplings, single-factor repeated measures ANOVAs were subsequently run for all metrics, computed by pooling data for a given coupling scheme across motion profiles and initial motion directions. Confirming our second experimental hypothesis, we found that binaural bipolar GVS amplifies and attenuates perceptions of physical roll tilt, dependent on the positive and negative GVS couplings respectively. Moreover, we find that the extent of amplification and attenuation is dependent on the specific GVS waveform coupling. To compute effect sizes, Glass’s **Δ** was used for comparisons to the control (i.e., No GVS condition), and Hedge’s g was used in alternative comparisons.

For the Mean Absolute Tilt Perception metric, the single factor repeated measures ANOVA indicated a difference between the seven coupling schemes (F(6,60) = 13.07; p<0.001). On average, the Mean Absolute Tilt Perception metric yielded larger values for positive GVS coupling schemes (velocity = 1.65 ± 0.28 (mean±SD in deg); joint = 1.98 ± 0.43; angle = 1.89 ± 0.34) and smaller values for negative coupling schemes (velocity = 1.54 ± 0.38; joint = 1.37 ± 0.21; angle = 1.37 ± 0.34). Compared to the No GVS condition (1.52 ± 0.13), the Mean Absolute Tilt Perception metric reveals amplifying and attenuating effects respectively (Fig 1C). Post hoc tests with Bonferroni corrections comparing each coupling scheme with the No GVS condition showed significance for only the positive joint (t(10) = 2.5184; p = 0.032; **Δ** = 2.5) coupling scheme. Post hoc tests between the positive and negative versions of each coupling scheme showed significance between joint couplings (t(10)>4.587; p<0.001; Hedges’ g = 1.79) and angle couplings (t(10) = 3.413; p = 0.008; g = 1.48), but not pure velocity couplings. Subsequent ANOVAs between just the three positive and negative coupling schemes showed significance between the positive and approached significance between the negative coupling schemes (F(2,20) = 6.644; p = 0.006 and F(2,20) = 2.794; p = 0.085 respectively). These results support the earlier finding that perceptions depend on the GVS waveform and additionally reveal that the specific GVS waveform impacts the magnitude of amplification and attenuation. Comparing coupling schemes, including roll tilt as part of the coupling scheme produced stronger amplification/attenuation than the roll velocity couplings.

Reinforcing the finding that GVS amplifies and attenuates perceptions of physical roll tilt, Perceived/Actual Tilt Slope yielded steeper slopes for positive (amplifying) couplings (velocity = 1.02 ± 0.20; joint = 1.27 ± 0.29; angle = 1.22 ± 0.28; No GVS = 0.90 ±0.14) and shallower slopes for negative (attenuating) couplings (velocity = 0.70 ± 0.27; joint = 0.53 ± 0.48; angle = 0.49 ± 0.37) (Fig 1D). Repeated measures ANOVA indicated a difference between coupling schemes (F(6,60) = 19.7; p<0.001). Post hoc tests with Bonferroni corrections comparing each coupling scheme to the No GVS condition approached significance for the negative angle (t(10) = 2.215; p = 0.0516; **Δ** = -1.17) coupling scheme. Post hoc tests between the positive and negative versions of each coupling scheme showed significance between joint couplings (t(10)>4.587; p<0.001; g = 1.86 and angle couplings (t(10)>4.587; p<0.001; g = 2.23). Subsequent ANOVAs between just the three positive and negative coupling schemes showed significance between the positive and approached significance between the negative coupling schemes (F(2,20) = 10.9; p< 0.001 and F(2,20) = 2.69; p = 0.092, respectively).

### GVS-evoked canal afferent dynamics explain altered roll-tilt perceptions following central processing

**Fig 2:**
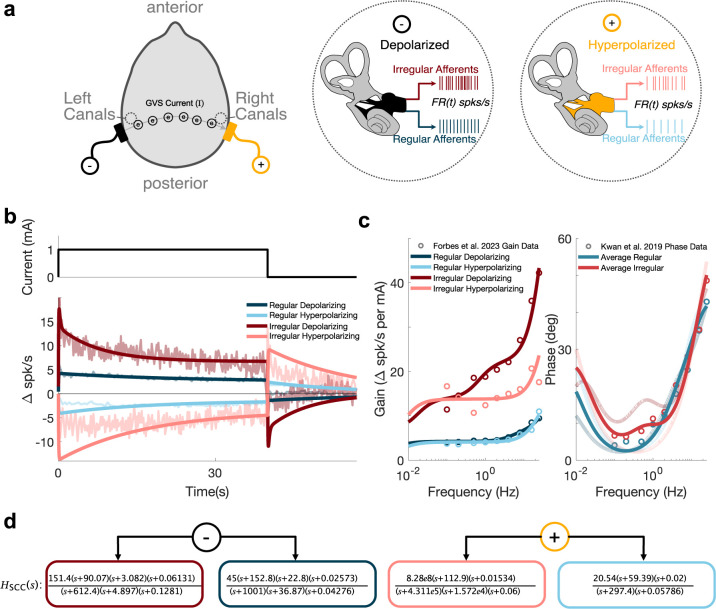
Providing an extension of the vestibular afferent transfer functions evoked by GVS to frequencies beneath 0.1 Hz via the essential works of Kwan et al. [29] and Forbes et al. [28]. **a**, Depiction of binaural bipolar GVS’s depolarizing and hyperpolarizing effect. The cathode side (negative terminal) increases irregular and regular firing rates (i.e., depolarization). Conversely, the anode side (positive terminal) decreases firing rates (i.e., hyperpolarization). **b**, Resultant firing rates following a 1 mA step current input and subsequent termination of signal (solid lines). The empirical population firing rates originating from Forbes et al. [28] (located in Fig 3A) are overlayed with corresponding colors. The lower portion of the transfer function was fit to match the step response in the 0-40s range. Note that the step response afferent firing rate data stems from a different population of afferents than those used to generate the empirical data in panel b, likely contributing to some overshoot of the transfer functions during the simulated step response. **c**, The gain and phase responses of the extended transfer functions, which more closely match the phase data from Kwan et al. [29] near 0.1Hz for both regular and irregular afferents. For comparisons to the phase data, which was not segregated for anodal and cathodal stimulation types, averages of the anodal and cathodal transfer function phases were taken. Cathodal and Anodal phase responses are shown in the background with consistent colors to the gain response curves. **d**, The four irregular/regular cathodal/anodal transfer functions, which now span a greater frequency range thus enabling modeling of both DC and dynamic (up to 25 Hz) GVS evoked firing rates.

A computational model of GVS’s influence on self-motion perception offers characterizing and predicting the effect of GVS on roll tilt and other motion percepts due to any arbitrary GVS or motion waveform. To realize this model, we first provide new model relationships describing the influence of GVS on the signals transduced by the semicircular canals (Fig 2A). Currently, there have been no direct recordings of human vestibular afferents, so we adopt the rhesus macaque vestibular afferent unit recordings as a proxy (convolved into a firing rate), provided by the recent works of Kwan et al. [29] and Forbes et al. [28]

First, we extend these existing transfer function approaches [28, 29] to capture the impact of GVS current on semicircular canal afferent firing rates, both for DC (Fig 2B) and sinusoidal currents (Fig 2C). Our transfer functions (Fig 2D) leverage the sub-0.1Hz frequency content inherent to the step response dynamics to characterize linear transfer functions more broadly at lower frequencies for both depolarizing/hyperpolarizing stimulation and regular/irregular afferents. Leveraging these extended transfer functions, we mechanistically model the influence of GVS on the human percept of tilt through peripheral modulation of the signals transduced by the semicircular canals across frequencies (roughly spanning 0.01-25Hz and critically, during DC stimulation).

**Fig 3:**
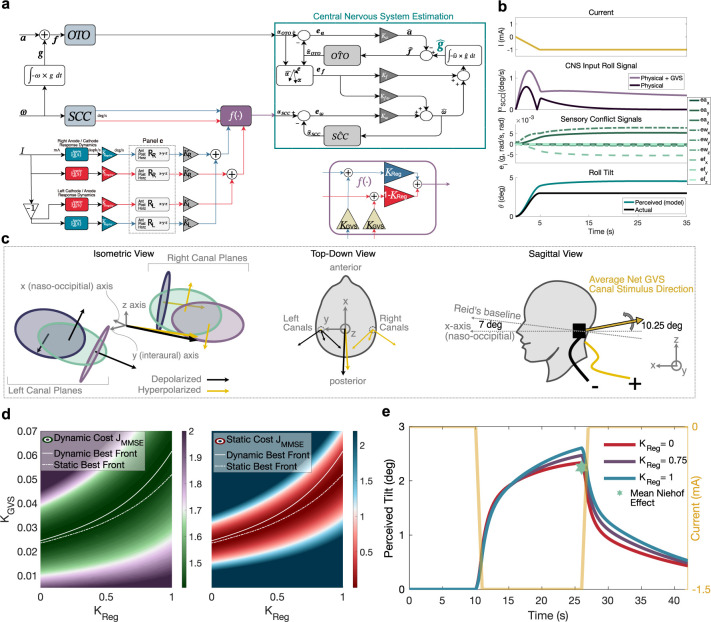
Peripheral modulation of the canal afferents is coupled with an observer model of human motion perception. **a,** Both physical motion-evoked and GVS-evoked afferent responses are combined for input into central nervous system estimation. **b**, Sequential model computations using the optimal point at ***K***_***Reg***_ **= 0 and *K***_***GVS***_ **= 0.0245. c,** GVS stimulates the anterior (blue), posterior (purple), and horizontal (green) canals, and their net signal is realized in the observer orthonormal reference frame as the linear combination of these individual vectors. **d,** Model training optimal fronts are displayed using both our dynamic experimental paradigm’s training dataset (solid white line) and Niehof et al.’s **[22]** static DC GVS paradigm (dashed white line), overlain on the mean squared error cost computed using the dynamic training dataset (green-purple contour) and separately on the mean squared error cost computed using the average effect observed by Niehof et al. **[22]** (see the Discussion for potential non-linearities as higher GVS current amplitudes are applied). Considering each paradigm alone, an optimal front exists where no single solution best explains the empirical dataset for a single paradigm. However, congruency between both paradigms is best achieved when ***K***_***Reg***_ **= 0**. Conversely, when *K*_*Reg*_ = 1, the best fit of the dynamic training dataset incurs a large cost in the static dataset (while the inverse is not as extreme). **e,** To demonstrate this finding, three model simulations are conducted using three points on the dynamic best front corresponding to ***K***_***Reg***_ **= 0** (irregular only dynamics) in red, ***K***_***Reg***_ **= 0.75** (mixed 3:1 regular:irregular dynamics) in purple, and ***K***_***Reg***_ **= 1** (regular only dynamics) in blue. These simulations are compared to the mean Niehof et al. **[22]** effect of DC current (green star), which was used to compute the static front.

To model how the CNS processes peripheral signals into percepts of self-orientation and self-motion, we utilize the commonly adopted observer framework to model central processing and sensory integration of semicircular canal and otolith pathways (Fig 3A and 3B). The observer model of spatial orientation perception first simulates how passive physical head movements result in vestibular afference. Following this, perceptions of angular velocity, linear acceleration, and tilt are produced through weighted comparisons of vestibular afference to expectations of vestibular afference (i.e., sensory conflict). These expectations are computed from internal models of sensory dynamics and kinematic relationships.

We next hypothesize that the effect of GVS on vestibular afferents is additive to changes evoked by physical motion and additionally hypothesize that the same central mechanisms that process physical vestibular afferents are used with GVS-modulated afferents. Further, while GVS is known to impact individual otolith afferents [28, 29], the net influence of GVS on gravito-inertial cues transduced by the system of otolith afferents has been hypothesized and estimated to be near zero given the mirrored orientation of hair cells innervating the otolithic macula across the striola [30]. We adopt this third hypothesis for our modeling, leaving only the influence of GVS on the signals transduced by the canals, with relative depolarization and hyperpolarization components in the primary axes/planes of each of the three left and right canals (individual and net canal signal directions evoked by GVS for positive current are depicted in Fig 3C). Notably, while we model the effect of GVS as linear and additive on canal sensory transduction, we do not assume linear effects of GVS on resultant tilt perceptions, which are influenced by central processing. With this framework, our modeling approach of GVS’s influence on the canal afferents alone is capable of explaining tilt amplification and attenuation in the presence of physical motion. This is achieved by modeling how the CNS compares peripheral signals, altered by GVS, to the CNS’s expectation of those signals obtained via internal models of vestibular sensor dynamics and kinematic relationships. Subsequently, our model reveals how errors in expectation (sensory conflict) are altered by GVS, leading to amplified/attenuated percepts of gravity’s direction and thus tilt (Fig 3B).

### Irregular afferent dynamics alone best explain observed self-orientation perceptions

Because GVS-evoked regular and irregular afferent dynamics differ from those evoked by physical motion, we investigated the roles of each afferent channel in human orientation perception by assessing if model fit depends on afferent regularity. Two free parameters are introduced in our model: ***K***_***GVS***_ and ***K***_***Reg***_ (where ***K***_***Irreg***_ **= 1−*K***_***Reg***_). The former (i.e., GVS gain) relates scaling differences (e.g., differences in anatomies, electrode configurations, model unit conversions, etc.) between different experiments in rhesus macaque and human afferent dynamics evoked by GVS, and the latter (i.e., regular afferent channel gain) describes the canal regular afferent channel contribution to perception (vs. irregular afferents). Training the model considering four of the six GVS coupling schemes (tilt-coupled GVS and velocity-coupled GVS, both positive and negative, were used for training the model), there are non-unique best fits, revealing an optimal front (Fig 3D). To resolve these underdetermined optimal solutions into a unique set, we introduce a second paradigm: DC GVS during no physical motion (i.e., static GVS) from Niehof et al. [22]. A second optimal front was computed by simulating this experimental paradigm and comparing the model tilt gain to the average impact of GVS current on roll tilt perception found by their participant population (~1.5 deg/mA). A best fit considering both paradigms occurs when ***K***_***Reg***_ = 0 with the optimal fronts diverging from one another when ***K***_***Reg***_ increases beyond 0. Hence, the irregular canal afferent channel alone best predicts perceptions of tilt in the presence of GVS when considering DC GVS and dynamic GVS waveforms (comprised of 0.07–0.36Hz frequencies; Fig 3D and 3E).

### Model predictions are more predictive than physical tilt and are equally predictive compared to unseen test data

**Fig 4:**
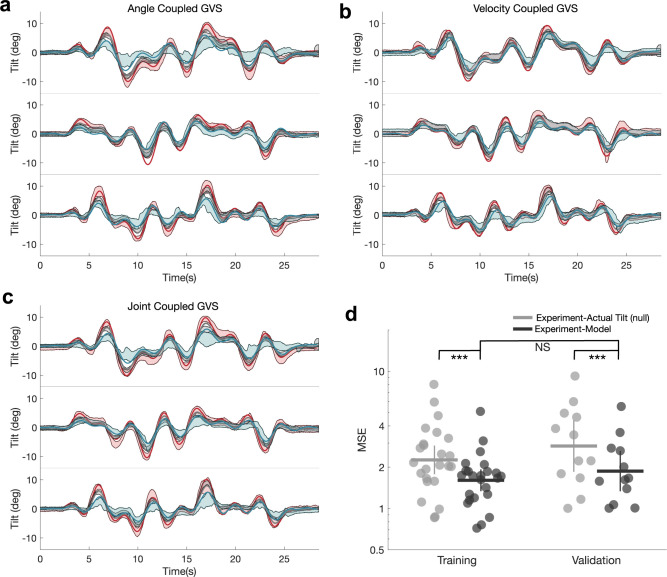
a-c, Model predictions (solid lines) are shown for amplifying (red) attenuating (blue) and no-GVS (grey) conditions, averaged across all participants and repeat trials of mirrored motions using the optimal point at *K*_*Reg*_ = 0 and *K*_*GVS*_ = 0.0245 Shaded regions correspond to the SEM bounds of our empirical data (N = 11). **a**, Model predictions using tilt angle coupled GVS. **b,** Model predictions using tilt velocity coupled GVS. **c,** Model predictions using joint coupled GVS (the test dataset). **d,** Comparisons of model performance (inversely related to the evaluation metric, MSE, computed against the empirical data) compared to a null hypothesis (that angle tilt angle explains the empirical data). The model performed significantly better than the null in both the training (***p<0.001) and test (**p<0.001) datasets. No significant differences in model performance were found between the training and test datasets.

With the model trained, we sought to evaluate its predictive capability. Model predictions were evaluated both against the training (Fig 4A and 4B) and test (Fig 4C) datasets (i.e., the joint coupled GVS scheme couplings, both positive and negative, were not used in the training of the model’s free parameters). We quantitatively assessed model predictions as against a null hypothesis that our model cannot better predict perceptions than physical tilts alone. For this, MSE was computed between the mean empirical perceptual reports and our model predictions (Experiment–Model), as well as between the mean perceptual reports and the provided physical tilt (Experiment–Actual Tilt (null)). This was conducted for each amplifying, attenuating, and No GVS condition for the training dataset (24 combinations of GVS and motion) and for the test dataset (12 combinations of GVS and motion), provided in Fig 4D. We found the model to have significantly lower MSE than actual tilt, evaluated on both the training dataset (t(23) = -3.93; p<0.001) and the test dataset (t(11) = -3.92; p<0.001). For our computational model, there was no difference between the training and test fits using MSE (t(34) = -0.93; p = 0.36), suggesting generalizability.

### An assortment of elicited perceptions are predicted, dependent on gravity and individual differences

Next, we asked how perceptions of roll-tilt evoked by GVS may be modulated from various physical stimuli, and we sought to characterize how differences in susceptibility to GVS in our sample population might be realized in these scenarios. To first capture individual differences to GVS, both the training and test datasets were used to compute participant-specific GVS gains, the model gain producing the lowest MSE between model predictions and the entirety of a participant’s empirical perceptual reports during the GVS trials. We report the distribution of participants’ individualized gains on a scale that is normalized by the best-fit ***K***_***GVS***_ during training (0.0245) while setting ***K***_***Reg***_ = 0. This metric serves as a robust means to capture the dynamic effects of GVS on perception (i.e., ***K***_***GVS***_ = 0 indicates no effect) across all GVS waveforms and physical motion profiles using a scalar metric. Our population’s distribution was found to be normally distributed (Shapiro-Wilks test of normality: p = 0.37) with ***μ*** = **1.03** and ***σ*** = **0.41** (with individual values provided in Table A in S3 Text). This distribution is significantly non-zero when analyzed by a one-sample two-tailed t-test (t(10) = 8.33; p<0.0001) and all of our participants were found to have positive GVS gains, meaning that GVS produced a significant effect and altered roll tilt perceptions for all participants in a directionally consistent manner. This statistical outcome reinforces our empirical findings using simple model agnostic metrics (Mean Absolute Tilt Perception and Perceived/Actual Tilt Slope).

**Fig 5:**
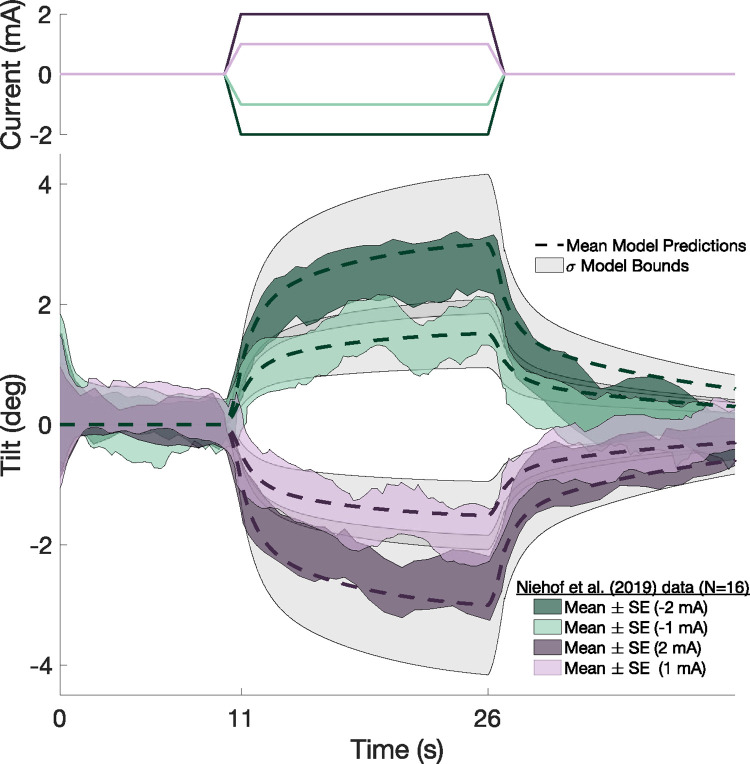
Mean model predictions (dashed lines) and *σ* bounds (grey shaded regions) generated by simulating the DC paradigm in Niehof et al. [22] are compared to their empirical data, unbiased by an average reporting bias of -0.43deg and time delay of 0.6s. Corresponding model inputs (current without physical motion) are shown above the perceptions and model predictions. The model mean predictions largely lay within the SEM bounds of the 16-participant mean perceptions during an upright (i.e., no physical roll tilt) DC GVS stimulation paradigm. While DC GVS creates near-instantaneous changes in afferent firing rates (Fig 2), resultant perceptions of tilt for the onset of DC GVS stimulation contain delayed transient dynamics which eventually approach a steady state. Following the termination of DC GVS stimulation, perceptions of tilt demonstrate exponential decay with two dominant time constants. Our model predictions capture the time course of these temporal dynamics.

**Fig 6:**
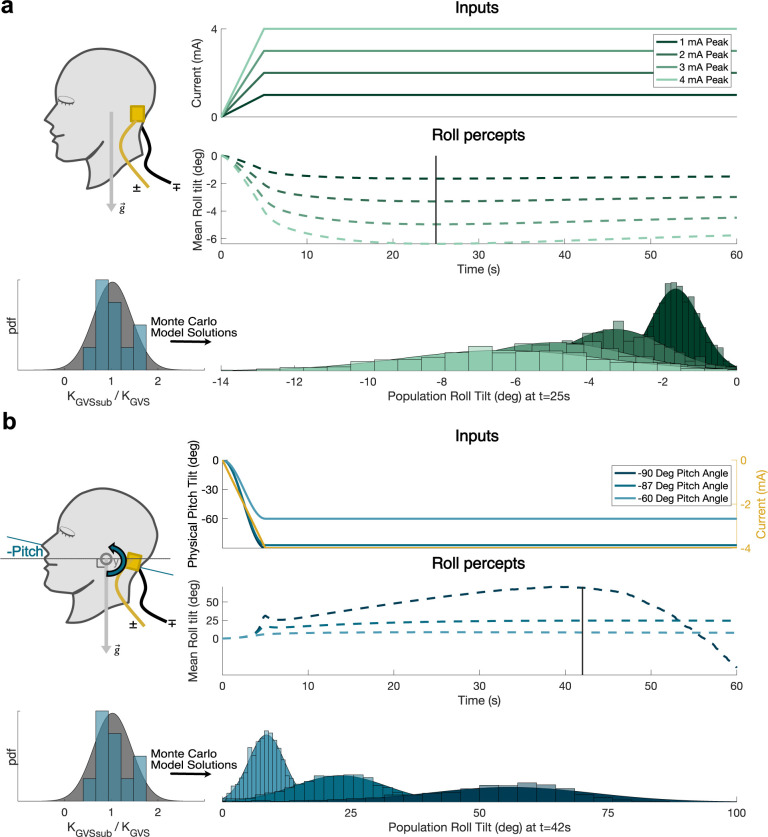
Mean and population distribution roll tilt perception predictions for new, testable paradigms without empirical data. Population perception distributions are formed through 1,000 Monte Carlo simulations, sampling from the population GVS susceptibility distribution. **a,** 1, 2, 3, and 4**mA** DC GVS applied in the absence of physical motion while upright. At a peak mean perception occurring at t = 25s, population distributions for the four DC GVS cases are shown, all with equal CVs. When upright without physical motion, a mean effect of -1.6deg/mA is predicted at 25 seconds of DC stimulation. **b,** 4**mA** DC GVS applied in conjunction with quasistatic pitch tilts. At a peak mean perception occurring near t = 42s, population distributions for the three DC GVS cases are shown, all with equal CVs. Pitching back to 90 degrees allows for delivering greater roll tilt perceptions than when upright (shown in a) or at intermittent pitch angles. The effect is nonlinear, demonstrated by the three pitch tilts shown here.

Characterizing individual susceptibility to GVS additionally allows us to generate uncertainty bounds on our mean model predictions by directly sampling from our population distribution via Monte Carlo simulations. We compare our model predictions with resultant ***σ*** bounds to the SEM bounds reported in Niehof et al. [22] (Fig 5). To demonstrate new utility afforded by this model using our findings, we extend our predictions with resultant population distributions to paradigms without empirical data for the case of direct current stimulation up to 4**mA** (Fig 6A) and direct current stimulation at 4**mA** with varying pitch tilt profiles (Fig 6B). In doing so, we find that the population coefficient of variation is predicted to be constant across GVS applications, equal to the coefficient of variation of $p_{K_{GVS}}$, ***CV* =** 0.40, with an individual’s underlying GVS susceptibility determining the magnitude of their predicted tilt perception. This predicts larger inter-individual variations in tilt perception produced by GVS when larger amplitude currents are applied.

## Discussion

In examining perceptual data of humans during dynamic GVS waveforms in the presence of dynamic physical motion, we explored multiple GVS coupling schemes with the expectation that all conditions with GVS would alter roll tilt perception. We further hypothesized that positive current coupling would lead to an amplifying effect and negative current coupling would lead to an attenuating effect, and that coupling GVS current to different motion parameters may change the magnitude of the amplification or attenuation. Our results support these hypotheses.

Notably, angle coupled GVS had the greatest amplification/attenuation effect on average while velocity coupled GVS had the least (considering either metric). Because the semicircular canals transduce angular velocity, one might expect that GVS stimulation coupled with angular velocity would most amplify/attenuate the semicircular canals’ transduction of angular velocity and lead to the most amplified/attenuated net tilt perception. However, our results in this study indicate that the instantaneous polarity and magnitude of the GVS administered is a better predictor of how tilt perception will be altered during low-frequency whole-body roll tilts (i.e., positive current tends to shift tilt perception toward the cathode side; best illustrated in Fig 1B between 16-20s for the amplifying GVS and 8-12s for attenuating GVS). Using GVS coupled to angular velocity was recently explored in an attempt to produce post-roll flight illusions in stationary participants using GVS **[31]**, and our data suggest that alternative couplings can produce a stronger sensation of illusory tilt. It has been hypothesized that the CNS leverages an internal model of gravity’s direction to perceive just tilt under net semicircular canal modulation from GVS **[22, 23]**, though a quantitative model of CNS processing of vestibular sensory information modulated by GVS has not been explored.

We constructed a model, quantitatively capturing the influence of GVS on spatial orientation perception, built on three primary hypotheses that are examined herein. The first is that the net effects of GVS on the left and right otolith systems, is near zero for low levels of GVS current (such as those used in this study; ≤**4*mA***). The second is that GVS-induced afferent modulations are linearly additive to those produced from physical angular rotations. The third hypothesis is that the central processing of canal-otolith integration employed during naturalistic physical motion are also employed during GVS application. Through this modeling approach, with a single GVS effect scaling parameter (***K***_***GVS***_), our model was able to capture our empirical datasets of self-orientation, including unseen test data, thus lending credibility to these hypotheses while additionally providing the utility to predict quantified perceptions of self-motion due to arbitrary 6 DOF motion stimuli and GVS for the first time. Together, these data and mechanisms reveal insights into the peripheral modulation of motion signals due to GVS as well as the neural substrates responsible for spatial orientation perception in humans.

From our computational simulations, we found that GVS alterations of rotation rate information transduced by the canals lead to altered perceptions of tilt, explaining our experimental findings of tilt over and underestimation. This result bolsters the ‘GIF resolution hypothesis’ that canal cues are utilized by the CNS to determine gravity’s direction **[32, 33]**. In accordance with Einstein’s equivalence principle **[34]**, stimulation from gravity and linear acceleration are indistinguishable from otolith cues alone. The observer model of self-orientation perception hypothesizes the CNS resolves tilt-translation ambiguity by utilizing an internal model capturing the following relationship:

$$\overset{\wedge }{g}(t)=\int \overset{\wedge }{g}(t)\times \overset{\wedge }{\omega }(t)dt.$$ (1)

Critical for GVS applications, this relationship enables eliciting altered perceptions of tilt (dependent on the perception of gravity, $\overset{\wedge }{g}\in R^{3}$) by modifying semicircular canal signals without modifying otolith cues. In turn, these modified canal signals affect the neural representations angular velocity, $\overset{\wedge }{\omega }\in R^{3}$, which are fed through the CNS’s internal models used to disambiguate otolith cues (i.e., tilt/translation discrimination), recently proposed to exist in the Nodulus and Uvula lobules (i.e., the otolith vermis: nodules X and IX) of the cerebellar vermis [35]. Because the direction of $\overset{\wedge }{g}$ changes with incoming vestibular measurements, all three components of $\overset{\wedge }{\omega }$ influenced by GVS (depicted in Fig 3C) influence the percept of roll tilt (e.g., Merfeld et al. [36] demonstrated how earth-vertical yaw canal cues can affect roll tilt perception when sitting upright). Our computational model quantifies how peripheral alteration of the semicircular canal cues via GVS results in a modified perception of angular velocity and subsequently modified perception of tilt in humans by way of the internal models associated with tilt/translation discrimination.

Additionally captured by the observer model, tilt estimates are continuously influenced towards the GIF direction sensed by the otoliths (the ***K*_*f*_** pathway in Fig 3A). This known phenomenon during physical motion (referred to as “somatogravic feedback”) can result in misperceptions of tilt due to large translational accelerations [37]. Crucial for GVS, this effect prevents continuous angular velocity cues from resulting in perpetually increasing roll tilt perceptions and instead explains the empirically observed effect of tilt over/under estimation reaching a steady state over time when physically upright. Reducing the component of the GIF perpendicular to the rotation axis (such as simulated in Fig 6B), enables more capability of producing tilt perceptions, using GVS (and using GVS in a partial gravity environment should modify the effect of GVS even further). Substantiating this prediction, humans have reported larger rotations following tilting their heads [38] as well as sensations of whole-body rotation while lying on their backs during GVS [39]. In another study, Khosravi-Hashemi et al. [23] made qualitative predictions of perceptions of linear acceleration and angular velocity based on different static head positions using a model of central processing of vestibular information. However, because they neither modeled canal afferent activation nor implemented a perceptual task to quantify perceptions of motion, they could not make quantitative predictions of dynamic self-motion perception, nor validate their model. Here, we leverage an improved understanding of tilt-translation discrimination during GVS using the observer model as well as GVS’s influence on canal afferent firing rates to explain our novel empirical findings of altered orientation while also enabling broad, quantifiable predictions of self-motion. Our study explains these previously observed qualitative effects with quantifiable predictions and is compatible with prior findings.

Because GVS differentially alters regular and irregular afferent neurons in a way that is inconsistent with stimulation during physical motion, our model enables us to evaluate which afferent channel (irregular vs regular) best explains empirical perceptions when utilizing a firing rate-based afferent coding approach. We found that the peripheral firing rate dynamics of the irregular afferents, with their shorter exponential-decay time constants, better explain both dynamic and DC GVS paradigms than regular afferent dynamics (though fits were only somewhat worse with small contributions of regular afferents, as seen in Fig 3D). Related to this finding, vestibular-only (VO) neurons in the vestibular nuclei are thought to predominantly receive input from irregular afferents [1, 40] and display similar spike-timing precision to irregular afferents [41]. Downstream, VO neurons project to the vestibular cerebellum and thalamus, brain regions associated with self-motion perception.

The primary attribute of the irregular canal afferents that makes them best suited to explain both dynamic current and direct current GVS stimulation is their decaying spike rate dynamics under DC stimulation, captured with the extended canal transfer functions we presented here (Fig 2). These dynamics explain the Niehof et al. [22] static dataset’s behavior during and immediately after DC GVS stimulation (Fig 5) while providing congruency with our dynamic GVS and physical tilt dataset (Fig 4A–4C). While this electrophysiological attribute of the irregular afferents stems from the firing rate dynamics examined in rhesus macaques, this attribute likely exists in humans as well due to the similar afferent morphologies between these two primates. As a consequence of morphology, differing interspike trajectories drive differences between irregular and regular discharge regularities [42], which is likely responsible for the differing temporal dynamics.

Previous rate-based modeling efforts have suggested that a 3:1 ratio of regular to irregular signals contributes to perceptual pathways [24, 28] based on the relative population of these channels innervating the chinchilla’s canals [43]. Contrarily, it has also been speculated that regular and irregular afferents contribute to different pathways (reviewed in [1]). Using GVS, it has been found [44] that acutely ablating the irregular afferents in squirrel monkeys (which is not currently possible to confirm in human participants) does not significantly affect the vestibular-ocular reflex, suggesting that regular afferents dominantly contribute to this pathway. Likewise in rhesus macaque subjects, recent vestibular prosthesis research found that stimulation mappings that best match regular afferent behavior best restored the temporal dynamics of VOR function [45].

The final implication of irregular afferents contributing to perception arises from the recent discovery that modulation of firing rates in vestibular efferent neurons preferentially modulates firing rates in vestibular irregular neurons [46]. Future works should explore the possibility of modulating irregular afferents, and resultant perceptions of self-motion, through stimulation of efferent neurons. Particularly, upstream neurons in the brainstem may enable generating augmented perceptions that avoid unwanted simulation of potential regular afferent mediated pathways (such as the VOR), and such studies may be able to further probe the role of irregular, regular, and efferent neurons.

Concerning our GVS gain, ***K*_*GVS*_** = 0.0245 reveals the magnitude of the GVS effect. Notably, this value is influenced by the unit conversion from ***Δspk*/*s*** to deg/s (1.85) chosen for the irregular pathway (see Methods). A more direct comparison can be made by examining the peak virtual angular velocity magnitude (deg/s) evoked by GVS per mA of applied current. Using ***K*_*GVS*_** = 0.0245 with ***K*_*Reg*_** = 0, we find that GVS evokes a peak population mean canal signal of angular rotation about the net GVS vector of ~1 deg/s per **mA**. Critically, our model predicts the dynamics of this rotation rate effect, which follow the afferent dynamics described by the anode/cathode transfer functions. A prior qualitative modeling effort (lacking quantitative validation) from Khosravi-Hashemi et al. [23] used an estimated static effect of 2.5 deg/s per **mA** (indirectly estimated based on direction recognition threshold data [47] and sub-threshold nulling data [21]). Thus our model effort builds on the qualitative predictions (without physical motion) of Khosravi-Hashemi et al. [23], and by adding physical motion pathways, an empirically driven effect of GVS on canal signals, and afferent dynamics, allows for quantifying the dynamic tilt effect observed in our dataset, the Niehof et al. [22] dataset, and also other, not yet tested perceptions of self-motion and self-orientation with and without physical motion. The additional work of Chen et al. [24] used a similar effect of 0.37 **mA** per deg/s (2.7 deg/s per **mA**), however, comparisons to their model are not directly possible because they used inverted physical dynamics to convert between ***Δspk*/*s*** to deg/s without central processing and an alternative summing of physical and GVS signals, which we avoid here.

Our multi-paradigm, data-informed, full 6dof model enables quantified computational assessments of GVS’s impact on perceptual responses in humans for the first time thus unlocking the biomedical engineering capabilities of GVS. This utility is explored in Fig 6A and 6B across two separate untested paradigms. We predict a population distribution of roll tilt perceptions for direct current stimulation up to 4**mA** (Fig 6A), and during physical pitch tilts (Fig 6B). We note that we have assumed a linear transfer relating GVS current applied to the change in afferent firing rate, based upon afferent recordings in which only ±1**mA** of GVS was applied. Thus, our simulations are extrapolating when applying current amplitudes of up to 4**mA**. Future afferent recordings with higher current amplitudes could validate the linear transfer function assumption or inform fitting a non-linearity.

Concerning flight, this model lends to developing neurovestibular illusion ground paradigms, which otherwise can be lethal and mission compromising when experienced during operations. Recent works have highlighted the need to develop such ground-based paradigms [15, 17, 31], yet it has remained unclear how peripheral vestibular stimulation can be leveraged to achieve desired perceptions. Additional utility exists considering electrode montages which depolarize or hyperpolarize both the left and right vestibular systems, by means of additional electrodes placed away from the mastoid processes. Adapting this model to this montage may enable predicting dominant sensations of pitch (hypothesized in [30] and suggested by human postural sway data [48]).

Stemming from intermediaries of perception, sensory conflict theory relates vestibular sensory mismatches to the development of motion sickness symptoms [49–51]. Fig 3B demonstrates how GVS modifies vestibular sensory conflict signals during passive motion, which leads to perceptions of head motion and orientation. Physiologically, sensory conflicts are thought to be analogous to VO neurons, which respond to the passive (i.e., unexpected) contributions of motion. The computational model of perception developed herein enables constructing current profiles that attenuate motion sickness symptoms in humans during passive motions when coupled to a computational model of motion sickness arising from vestibular sensory conflicts [49].

While our work is centered on perceptual pathways, future work can implement the framework used by this research endeavor, in conjunction with human VOR data during GVS and a computational model of the VOR pathway, to better understand the neural substrates contributing to the VOR in humans, vital for realizing the human vestibular prosthesis. Additionally, this model can be extended to study the influence of GVS in the presence of other sensory cues relaying self-motion and self-orientation information as well as active motion and when the head is unrestrained. In the case of the latter, contributions from postural responses evoked by GVS must be modeled to account for altered perceptions compared to the head-immobilized case while standing [52].

## Methods

### Ethics statement

This study was approved by the Institutional Review Board of the University of Colorado, Boulder (Protocol 22–0171). Fourteen participants signed the written informed consent documents, but only 11 (age: 24.75 (SD = 1.83), 4 female) produced usable data sets. One was excluded due to an early onset of motion sickness. The others were excluded due to failure to perform the SHH tasks. Participants reported no known vestibular dysfunction and refrained from consuming alcohol within 6 hours prior to the study. Participants were between the heights of 5’2” and 6’3” and weighed less than 225 lbs as required by the TTS motion device used in the study.

### Dynamic tilt experiment protocol

Participants were first outfitted with a 2x2” square sponge electrode on each mastoid which were held in place with a headband for all conditions (including the No GVS sham condition). Impedance was checked and found to be below 12kΩ for all participants. Participants received sample GVS profiles both with head restrained and unrestrained to ensure all equipment was working before proceeding with the experiment. For the experiment, participants sat upright in the Tilt Translation Sled (TTS), a motion device capable of providing combinations of tilt and translation, with their heads restrained by firm foam blocks on each side which were adjusted to fit the participant’s head. The lights were off to remove visual cues and white noise was provided to mask auditory cues from the device’s motors. During trials, participants were tilted about a head-centered roll tilt axis along a 30s pseudorandom sum of sines profile (±8°) with or without GVS stimulation, during which they continuously reported their sense of tilt with a subjective haptic horizontal (SHH) task. A SHH task was utilized, rather than a subjective visual vertical task to avoid potential effects of ocular torsion responses produced by GVS that might confound a subjective visual vertical task [53]. Two-way audio communication and one-way video allowed operators to make sure participants were ready at the start of each trial and to monitor participant motion sickness. Participants reported their level of motion sickness on a scale of “none”, “slight”, “moderate”, or “severe” after every other trial for reports of “none” and every trial following an affirmative report of motion sickness. Two consecutive reports of “moderate” resulted in an early termination of the study for one participant.

### Data acquisition of dynamic tilt perception in humans

A subjective haptic horizontal task was used to measure tilt perception continuously throughout the trial [54]. This was implemented via a bar mounted to a potentiometer, which rotated with participants’ whole-body tilt. Participants held the bar between their fingers and were instructed to adjust its orientation to keep it aligned with their perception of the horizontal. For perfectly accurate perception, the participant would move the bar such that it is continuously aligned with the true horizontal. Participants completed at least three training trials without GVS to familiarize themselves with the reporting task and were reminded on how to properly complete the task after every other trial.

### Experimental paradigms

Participants experienced six different motion profiles and seven different GVS coupling schemes. Motion profiles were based on three pseudorandom sums of sines with equal power frequency contents of: 0.07,0.18, and 0.31Hz; 0.07,0.25, and 0.33Hz; and 0.07,0.19, and 0.36Hz without phase shifts. Two full cycles of the 0.07Hz waveform were completed with the first and last half cycles including a sigmoidal ramp up/down. Each profile was then mirrored on the left/right (negative and positive roll tilt respectively) axis, creating 6 motion profiles. GVS coupling conditions included a No GVS sham condition, and GVS was either coupled to tilt angle, tilt velocity, or an average of the two normalized signals (joint coupled). To test the second hypothesis that the current direction dictates amplification and attenuation of roll tilt perceptions during physical motion, GVS was additionally coupled to run either positively (unlike sign) or negatively (like sign) with the physical coupling. Because we use a head-centric x-axis out the nose of the participant (i.e., positive roll corresponds to right ear down), positive coupling corresponds to a left anode right cathode GVS signal (negative current) when the physical motion characteristic is positive (hence GVS has unlike sign of the physical characteristic). The sign notation of GVS, where positive current corresponds to left cathode right anode current direction, was chosen to maintain consistency with existing literature [28–30].

The GVS waveforms were scaled so that the maximum current was +/-4**mA**. The GVS device was connected to the TTS such that initiating TTS motion also initiated the synchronized GVS waveform. Participants completed the No GVS case twice for a total of 48 trials. These trials were broken up into two blocks of 24 where each block contained a No GVS trial for all 6 motion profiles and one trial for each of the coupling schemes paired with only one of the left/right versions of the 3 base motion profiles. Combinations not contained in the first block were tested in the second block.

While the experimental paradigm only measured roll-tilt perception in the presence of physical tilts and GVS, one participant reported a lateral translation perception. Reports of GVS sensations of motion when upright are usually of tilt, however, stimulation typically occurs in circumstances where translation is unlikely. Here, the motion device we used to tilt participants in the experiment is also capable of translation. Although no translation occurred, this possibility and the lack of other sensory cues may have allowed the CNS to interpret GVS and physical stimuli as involving some translation, further supporting the need to consider central processing of GVS and physical motion rather than just peripheral vestibular modulation alone.

### Data processing and analyses

The raw SHH report was processed to account for differences in participant left/right biases, reporting delays, and deflection magnitude. To do this a single tilt angle correction bias (known to occur during similar perceptual tasks **[55]**), time delay correction (due to reaction times during the SHH task), and a gain-scaling factor (representing a participant’s tendency to overestimate/underestimate their perceived tilt using the SHH task) was computed for each participant based on their No GVS trials. After sequentially computing a participant’s reporting bias, delay, and gain, these adjustments were applied to the entirety of a participant’s data. Bias was computed as the bias that minimized the MSE of a participant’s raw reports and the actual tilt. Time delay was computed as the delay that minimized the MSE of a participant’s bias-adjusted reports and the actual tilt, and gain was computed as a gain on the bias and time-adjusted reports that minimized the average absolute difference in reports and actual tilt (so as to not penalize the rare opposite deflections of the bar during the SHH task and small tilts) when actual tilt was greater than 2 degrees (to minimize the influence of reporting during small tilts). Each participant’s correction parameters are provided in Table A in S1 Text.

Our two primary outcome metrics were derived from the adjusted SHH data: Mean Absolute Tilt Perception and Perceived/Actual Tilt Slope. Quantifying the magnitude of participants’ average perceived tilt during each condition, the Mean Absolute Tilt Perception is a simple measure of the variation of responses agnostic to the physical tilt, which we expect to increase with amplification and decrease with attenuation. Quantifying perceived tilt as a linear function across physical tilt angles, the slope of the actual and perceived tilt was computed (i.e., Perceived/Actual Tilt Slope) using least-squares regression. The Perceived/Actual Tilt Slope describes the magnitude of tilt over/underestimation. Both metrics were first checked for normality using a Shapiro-Wilks normality test. With only slight deviations in normality, we proceeded with ANOVA analyses due to their robustness against normality deviations.

### Statistical analyses

A repeated measures ANOVA was performed for each of the GVS effect metrics across each of the coupling schemes. This was followed by two-tailed paired t-tests between the No GVS condition and each of the other coupling schemes and two-tailed paired t-tests between each positive/negative coupling scheme pair. Effect sizes were computed for significant differences found via the follow-up t-tests. Glass’s Δ and Hedge’s G were used as appropriate for determining effect size in the case of comparisons to the control and comparisons between alternatives respectively **[56]**. Additionally, two subsequent, reduced ANOVAs were computed to compare just the amplifying and attenuating coupling schemes respectively. Bonferroni corrections for the maximum possible number of comparisons were used.

### Extending the afferent firing rate model dynamics of rhesus macaques

We build our transfer functions relating GVS current and canal afferent firing rates, based on those existing in the literature for rhesus macaques monkeys (Kwan et al. [29] and Forbes et al. [28]). The original canal transfer functions fit by Kwan et al. [29] provide population-level irregular and regular afferent responses (i.e., rate-coded firing rate, ***FR***(***t***)) for afferents stimulated by sinusoidal currents at 1**mA**. While these transfer functions do well in the 0.1-25Hz frequency range, the phase response begins to diverge at lower frequencies (around 0.1Hz) and they lack the exponential decay dynamics later provided by Forbes et al. [28] during cathodal (depolarizing) and anodal (hyperpolarizing) step current (direct current of 1mA) stimulation. Because of this gap, the Kwan transfer functions cannot be used to explain perceptual differences when DC current is applied and when sinusoidal (or sum-of-sines) current is applied below 0.1Hz. Forbes et al. [28] refit the gain data provided by Kwan et al. [29] separately for the portions of sinusoidal current corresponding to depolarizing and hyperpolarizing stimulations, but they do not provide these new transfer functions. Thus, we refit the separate depolarizing/hyperpolarizing transfer functions for the semicircular canals.

In doing so, we expand the functional range of frequencies that can be utilized by developing new population average transfer functions, building on these two prominent works of Kwan et al. [29] and Forbes et al. [28]. To not disturb the frequency characteristics of the transfer functions fit to the gain-phase data provided by Forbes et al. [28] in the 0.1-25Hz range, we selectively fit a transfer function with a gain upper limit (as ***s***→∞ where ***s*** = ***σ***+***jω***, the complex frequency variable) of unity, realized near 0.1Hz. Additionally required to represent the diminished steady-state afferent response noted by Forbes et al. [28], a lower limit (as ***s***→**0**) must be a constant less than unity. Together, the lower frequency response dynamics (***H***_***L***_(***s***)) is of the following form:

$$H_{L}(s)=\frac{s-z}{s-p}.$$ (2)

A full derivation of the form above is contained in S2 Text. The new transfer functions we contribute are the combined response of the fitted transfer function of the step response capturing the lower frequency response, ***H***_***L***_(***s***), and the upper-frequency response, ***H***_***U***_(***s***), found by fitting the sinusoidal depolarizing (cathodal) and hyperpolarizing (anodal) gain data, provided by Forbes et al. [28], with the average phase data, provided by Kwan et al. [29]:

$$H_{SCC}(s)=H_{L}(s)H_{U}(s).$$ (3)

The resultant step responses and gain-phase characteristics of ***H***_***SCC***_(***s***) for regular and irregular afferents are depicted in Fig 2.

### Interpreting canal firing rates evoked by GVS

These transfer functions describe the dynamics of how externally applied current results in a change in afferent firing rate from a resting discharge rate (producing units of ***Δspk*/*s***). In order to better understand the effect of current on the information transduced by the peripheral canal afferent pathways, we must convert changes in firing rate to units of angular velocity (as the semicircular canal afferents transduce angular velocity, which is processed by the central nervous system to produce perceptions). For GVS-evoked changes in afferent firing rates up to ± 50 ***Δspk*/*s***, canal afferent neurons respond linearly to changes in physical angular velocity [57]. Thus, we use gain-interpreters for the regular and irregular pathways, corresponding to the inverse gain of the passband region of canal activation from physical motion (in the frequency range of 0.2-2Hz) where canals afferents are thought to accurately transduce physical motion (and this range corresponds to the most experienced frequencies during natural human motion [58]). In this region, the behavior of canal afferents transitions from high pass-filtering to high-pass tuning [59]. Using physical motion to canal afferent firing rate transfer functions for the rhesus macaque (provided in [57]), which behave similarly to those of the squirrel monkey [59], the gain interpreters are 1.85 and 2.29 deg/s-per-***Δspk*/*s*** for the irregular and regular afferents respectively. However, the tuning parameter ***K***_***GVS***_ (defined below) accounts for differing values of the gain interpreters when considering human anatomy.

### Resolving the GVS-evoked afferent responses into an orthonormal basis

Similar to theoretical estimations first made by Fitzpatrick and Day [30], we calculate the virtual sensation of rotation in a head-centered orthonormal basis, denoted as the x-y-z coordinate system (subscript xyz). Here, the x-axis is aligned with the naso-occipital axis, the y-axis is aligned with the interaural axis, and z is perpendicular to these axes. We use the underpinning that the binaural bipolar GVS montage non-preferentially and equally stimulates the anterior, posterior, and horizontal canals (subscript APH) on a given side. The scalar magnitude of anterior-posterior-horizontal activation, ***ΔFR***(***t***)_***GVS***_, is determined by current signal and the transfer function ***H***_***SCC***_(***s***), which is dependent on if the local current is hyperpolarizing (anodal) or depolarizing (cathodal) and if the channel is regular or irregular (Fig 2A–2C). The following relationship is used to achieve this transformation:

$${\overset{\to }{\alpha }}_{GVS,xyz}^{R|L}(t)=R_{y}({N^{R|L})}^{-1}{\overset{\to }{\alpha }}_{GVS,APH}^{R|L},$$

$$\mathrm{and}\;{\overset{\to }{\alpha }}_{APH}^{R|L}(t)={\left[1\;1\;1\right]}^{T}\cdot \Delta FR{\left(t\right)}_{GVS}.$$ (4)

In the above equation, $N^{R|L}\in R^{3x3}$ is a basis comprised of unit normal vectors perpendicular to the planes of the average human anterior, posterior, and horizontal canals for either the right or left set of canals [60], and ${\overset{\to }{\alpha }}_{GVS}^{R|L}\in R^{3x1}$ contains the 3D rotation rate information evoked by GVS current in units of ***Δspk*/*s***. The additional rotation matrix, ***R***_***y***_, is a 7-degree rotation about the y-axis to align the x-axis of the GVS-evoked signal of virtual rotation from Reid’s Baseline down to the average naso-occipital axis. Excepting this final rotation, the approach dictated by the above equation is likely similar to other recent attempts to realize GVS-evoked signals in an orthonormal basis [24, 28] while not previously outlined.

Finally, we linearly combine the left and right afferent signals evoked by GVS through the following relationship:

$${\overset{\to }{\alpha }}_{GVS}=K_{R}{\overset{\to }{\alpha }}_{GVS,xyz}^{R}+K_{L}{\overset{\to }{\alpha }}_{GVS,xyz}^{L}.$$ (5)

We assume that the right and left vestibular contributions, ***K***_***R***_ and ***K***_***L***_ (and ***K***_***L***_ = **1**−***K***_***R***_) respectively, contribute equally (both are 0.5). This operation can be taken before or after combining with physical motion (next section/step) and is mathematically equivalent.

### Modeling the joint influence of physical and GVS stimuli

We represent the joint influence as a linear combination (depicted as the *f*(∙) block in Fig 3A):

$${\overset{\to }{\alpha }}_{SCC}=K_{Reg}({\overset{\to }{\alpha }}_{physical}^{Reg}+K_{GVS}{\overset{\to }{\alpha }}_{GVS}^{Reg})+{(1-K}_{Reg})({\overset{\to }{\alpha }}_{physical}^{Irreg}+{K_{GVS}\overset{\to }{\alpha }}_{GVS}^{Irreg}).$$ (6)

In the above equation, ${\overset{\to }{\alpha }}_{SCC}$ is the net 3D rotation rate information due to both GVS and physical motion (subscript physical). Given our participants reporting of no history of vestibular dysfunction, we assume that the physical afferent transductions of the left and right sets of canals (for both ${\overset{\to }{\alpha }}_{physical}^{Irreg}$ and ${\overset{\to }{\alpha }}_{physical}^{Reg}$) are equal, thus we utilize models of physical canal afferent transduction previously utilized within the observer model [25–27]. In the case of modeling unilateral vestibular loss with GVS for example, the physical canal signals would need to be altered and expanded such that:

$${\overset{\to }{\alpha }}_{physical}={K_{R}\overset{\to }{\alpha }}_{physical}^{R}+{K_{L}\overset{\to }{\alpha }}_{physical}^{L}.$$ (7)

The GVS-effect gain, ***K***_***GVS***_, serves as a means of tuning the model via training to account for linear differences in human and rhesus macaque anatomy and electrode configurations (that may amplify or attenuate the effect of GVS on changes in canal firing rates). Further, anatomical differences may also result in different passband gains (used as our angular velocity gain-interpreter). Finally, while efforts were made to suppress non-vestibular cues of verticality, this gain also includes other factors that impact tilt perception inherent to the empirical data. When fitting ***K***_***GVS***_ to individuals, this gain captures differences in the parameters of an individual’s particular observer model/CNS neural circuitry for spatial orientation perception.

The relative contribution of the regular afferent pathway to perception is denoted by the gain ***K***_***Reg***_∈[**0, 1**]. Complimentarily, the relative contribution of the irregular pathway is 1-***K***_***Reg***_. The set of free parameters for model training is comprised of ***K***_***GVS***_ and ***K***_***Reg***_.

### Modeling self-orientation perception with an observer CNS representation

The modulation of vestibular afferents alone is insufficient to describe human perceptions, which are dependent on central expectations of sensory afference, generated through internal models of sensory dynamics and of the physical laws governing head motion [25–27, 33, 61, 62]. Such is an ‘observer’ model of self-motion and self-orientation. Here we utilized the observer framework with model parameters outlined in Clark et al. [25], consistent with other recent models of human perception [49, 63–65] and informed by a host of passive motion paradigms [27, 36, 66–68]. As depicted in Fig 3A, the observer model was augmented such that canal sensory afferents were modulated by binaural bipolar GVS, and because GVS is transient, the internal models of end-organ dynamics were unmodified (i.e., the internal models are assumed to remain maladapted to GVS).

### Model training and validation procedure

We organized our empirical data into a training dataset, comprised of the angle-coupled and velocity-coupled perceptual responses, and a test dataset, comprised of the joint coupled perceptual responses for model validation. Our cost function was chosen to be the mean squared error computed between the model predictions and the mean perceptions in order to estimate the mean roll-tilt perceptions of the sample population over time (***J***_***MSE***_ contours in Fig 3D). Because the best fit found using dynamic data alone yielded an underdetermined set of optimal parameters (solid white line in Fig 3D), we paired our solution with the best fit of a DC GVS paradigm with no motion from Niehof et al. [22]. The best cost of the Niehof et al. [22] dataset alone (computed as the mean squared error of the average effect found at the end of GVS stimulation with our model’s prediction) also yielded an underdetermined set of optimal parameters. However, when paired together, a single set of optimal parameters was found that fit both datasets at ***K***_***Reg***_ = **0**, insensitive to the relative weighting between the two optimal fronts.

Following model training, we compared the performance of our model on the training dataset to that of our model on the test dataset, computing a mean squared error for all experimental conditions. Additionally, we compared the model performance to a null hypothesis that the perception could be explained by actual physical tilt alone. Two-tailed t-tests were conducted for both analyses. Statistical analyses were performed using log-transformations of the MSEs, which were found to be normally distributed (the un-transformed data was significantly non-normal using the Shapiro-Wilks test of normality).

### Quantifying individual differences

In finding no differences between our model’s predictions on the training and test dataset, we fit an individual GVS susceptibility factor metric, $K_{GVS_{Sub\{i\}}}/K_{GVS}\forall i\in \{1,\ldots ,N=11\}$, using each participant’s entire perceptual dataset during GVS (i.e., all positive and negative GVS trials during angle, velocity, and joint coupling schemes). A mean squared error cost function was chosen to estimate the mean of each participant’s GVS susceptibility as expressed within the confines of our experiment. We used a Shapiro-Wilks test of normality to confirm that the GVS susceptibility metric was normally distributed, and then we fit a normal distribution to our participants’ susceptibility metrics. One participant was revealed as an outlier with a normalized GVS susceptibility metric of 2.7, more than 1.5 times the interquartile range above the 3^**rd**^ quartile, making the distribution non-normally distributed. Upon visualization of this participant’s trials (participant 8), we noticed they often stopped adjusting their subjective haptic report for small tilts. Analogous to the reporting gain metric, refitting their data while ignoring portions when the actual tilt was under two degrees yielded a normalized GVS susceptibility metric of 1.7, which is within the normal distribution of the other participants.

## Supporting information

S1 Text Notes on participant raw data processing. (DOCX)

S2 Text Notes on the Lower Frequency Transfer Function Derivation. (DOCX)

S3 Text Notes on participant GVS effect gains. (DOCX)
