# Supplementary material for: Human perception of self-motion and orientation during galvanic vestibular stimulation and physical motion
Source: PLoS Comput Biol. 2024 Nov 18;20(11):e1012601. doi: 10.1371/journal.pcbi.1012601 (PMC11611259; doi:10.1371/journal.pcbi.1012601)
Supplement: S1 Text — (DOCX) [file pcbi.1012601.s001.docx]

# Notes on Participant Raw Data Processing

Each participant’s ground truth perception of tilt was assumed to be related to the continuous reports of the subjective haptic horizontal task via the following form:

$Perceived Tilt(t)=\left( SHH Angle\left( t+delay \right)+Bias \right)*Gain$,

where a single delay, bias, and gain existed for each individual participant. These computed values, assessed during the No GVS trials as outlined in the Methods section, are provided in the table below.

Table A. Individual participant time delays, left-right biases, and bar tilt gains were computed during the No GVS trials and used to non-preferentially adjust all experimental trials for a given participant.

| Participant ID | Time Delay (s) | L/R Bias  (deg) | Gain  (unitless) |
| --- | --- | --- | --- |
| 1 | $\mu=$0.30  [0.23 0.31] | $\mu=$ 1.51  [-0.48 3.28] | $\mu=$1.54  [1.20 1.93] |
| 2 | $\mu=$0.4320  [0.32 0.64] | $\mu=$ -0.64  [-3.26 1.46] | $\mu=$2.25  [1.70 2.69] |
| 3 | $\mu=0.27$  [0.19 0.39] | $\mu=$ 0.45  [-1.15 2.85] | $\mu=$1.78  [1.25 2.27] |
| 4 | $\mu=$0.39  [0.31 0.50] | $\mu=$ 1.73  [- 0.92 3.47] | $\mu=$2.91  [2.10 3.87] |
| 5 | $\mu=$0.30  [0.26 0.34] | $\mu=$ 0.35  [-2.9 2.69] | $\mu=$0.55  [0.34 0.84] |
| 6 | $\mu=$0.29  [0.18 0.45] | $\mu=$ -0.09  [-4.35 4.24] | $\mu=0.44$  [0.31 0.54] |
| 7 | $\mu=$0.48  [0.27 0.82] | $\mu=2.19$  [-0.41 6.5] | $\mu=$0.84  [0.56 1.28] |
| 8 | $\mu=$0.54  [0.36 0.71] | $\mu=$ 1.56  [-0.39, 4.72] | $\mu=$1.63  [0.90 3.32] |
| 9 | $\mu=$0.30  [0.19 0.54] | $\mu=$ -3.16  [-8.75 2.07] | $\mu=$0.65  [0.35 1.08] |
| 10 | $\mu=$0.28  [0.23 0.38] | $\mu=$ 1.38  [-0.95 3.58] | $\mu=1.98$  [1.34 2.90] |
| 11 | $\mu=$0.20  [0.15 0.26] | $\mu=$ 2.49  [-0.96 4.64] | $\mu=1.50$  [1.14 1.81] |
