# Supplementary material for: Human perception of self-motion and orientation during galvanic vestibular stimulation and physical motion
Source: PLoS Comput Biol. 2024 Nov 18;20(11):e1012601. doi: 10.1371/journal.pcbi.1012601 (PMC11611259; doi:10.1371/journal.pcbi.1012601)
Supplement: S2 Text — (DOCX) [file pcbi.1012601.s002.docx]

# Notes on the Lower Frequency Transfer Function Derivation

During cathodal and anodal step currents, the change in afferent firing rate from a resting discharge rate, $\Delta FR\left( t \right)$, was monitored in rhesus macaque subjects. For semicircular canal afferents, the four step responses (one for each combination of regular/irregular and hyperpolarizing/depolarizing current) are generally of the following form in the time domain:

$\Delta FR\left( t \right)=k[b+(1-b)e^{-t/\tau}]$

where $\tau>0$.

Here, $k$ is the initial response at t=0, and $kb$ (both positive constants) is the steady-state response following the step input. The firing rate decay noted during a step current input is modeled by the time constant, $\tau$. The initial response is mostly dictated by the upper frequency response, $H_{U}\left( s \right)$ (obtained by fitting a transfer function to the phase gain data provided by Forbes et al. (2023) in the 0.1-25Hz range) which behaves as a static gain at lower frequencies (i.e., $k$). Therefore, we consider the normalized response, $\frac{\Delta FR\left( t \right)}{k}$, to extract the form of lower frequency response.

We use the following relationship to solve for the lower frequency response from the step behavior:

$$Y\left( s \right)\mathcal{=L}\left[ \frac{\Delta FR\left( t \right)}{k} \right]=H_{L}\left( s \right)U\left( s \right).$$

where $s=\sigma+j\omega$ and $U\left( s \right)=\frac{1}{s}$, a 1mA step function input. The lower frequency transfer function, $H_{L}\left( s \right)$, can be solved for via the following:

$$H_{L}\left( s \right)= sY\left( s \right)=s(\frac{b}{s}-\frac{b-1}{s+\frac{1}{\tau}}).$$

Thus,

$$H_{L}\left( s \right)= \frac{s+\frac{b}{\tau}}{s+\frac{1}{\tau}}=\frac{s-z}{s-p}.$$

Here, $H_{L}\left( s \right)$ is of the form one-zero and one-pole, both negative values. This form is ideal for modifying the lower frequency response dynamics because the gain upper limit (as $s\to\infty$) of $H_{L}\left( s \right)$ is unity. Therefore, $H_{L}\left( s \right)$ can multiplied by $H_{U}\left( s \right)$ to yield the extended transfer function model so long as long as the pole corner frequency of $H_{L}\left( s \right)$ is sufficiently small enough to not interfere with frequency response dynamics captured by $H_{U}\left( s \right)$ above 0.1Hz.

The new, lower frequency extended transfer functions for the semicircular canals can be written as the following product:

$$H_{SCC}(s)=H_{L}\left( s \right)H_{U}(s)$$

To obtain $H_{SCC}(s)$ we first fit the transfer function $H_{L}\left( s \right)$ using the gain data provided in Forbes et al. (2023) and average phase data provided in Kwan et al. (2019). Subsequently, we fit the remaining zero and pole in $H_{SCC}(s)$ to the step response data. This approach was conducted for all four pairings of hyperpolarizing/depolarizing and regular/irregular afferent responses. The final transfer functions (plotted in Figure 2) are provided in the table below.

Table A. Details of the GVS-canal transfer functions

|  | $H_{L}\left( s \right)$ | | $H_{U}\left( s \right)$ | | $H_{SCC}(s)$ |
| --- | --- | --- | --- | --- | --- |
|  | # of zeros | # of poles | # of zeros | # of poles |  |
| Regular Depolarizing  (Cathodal) | 1 | 1 | 2 | 2 | $\frac{45\left( s+152.8 \right)\left( s+22.8 \right)\left( s+0.02573 \right)}{(s+1001)\left( s+36.87 \right)(s+0.04276)}$ |
| Regular Hyperpolarizing  (Anodal) | 1 | 1 | 1 | 1 | $\frac{20.54\left( s+59.39 \right)\left( s+0.02 \right)}{(s+297.4)(s+0.05786)}$ |
| Irregular Depolarizing  (Cathodal) | 1 | 1 | 2 | 2 | $\frac{151.4\left( s+90.07 \right)\left( s+3.082 \right)\left( s+0.06131 \right)}{(s+612.4)\left( s+4.897 \right)(s+0.1281)}$ |
| Irregular Hyperpolarizing  (Anodal) | 1 | 1 | 1 | 2 | $\frac{8.28e8\left( s+112.9 \right)\left( s+0.01534 \right)}{(s+4.311e5)\left( s+1.572e4 \right)(s+0.06)}$ |
