# Supplementary material for: Human perception of self-motion and orientation during galvanic vestibular stimulation and physical motion
Source: PLoS Comput Biol. 2024 Nov 18;20(11):e1012601. doi: 10.1371/journal.pcbi.1012601 (PMC11611259; doi:10.1371/journal.pcbi.1012601)
Supplement: S3 Text — (DOCX) [file pcbi.1012601.s003.docx]

# Notes on Individual Participant GVS Effect Gains

Each participant’s individual GVS susceptibility metric (${K_{GVS_{Sub\{i\}}}/ K}_{GVS} \forall i\in\{1,\ldots,N=11\}$), was estimated using a grid search that minimized the mean squared error of the empirical roll tilt perceptions (processed from their raw reports) compared to model predictions with $K_{Reg}=0$. These individual metrics are provided in the table below.

Table A. Individual participant GVS gains.

| Participant ID | $\frac{K_{GVS_{Sub}}}{K_{GVS}}$ |
| --- | --- |
| 1 | 0.65 |
| 2 | 1.65 |
| 3 | 0.85 |
| 4 | 0.65 |
| 5 | 1.15 |
| 6 | 1.05 |
| 7 | 0.50 |
| 8 | 1.70 |
| 9 | 1.40 |
| 10 | 0.75 |
| 11 | 0.95 |
